# On Wrinkling in Sandwich Panels with an Orthotropic Core

**DOI:** 10.3390/ma14175043

**Published:** 2021-09-03

**Authors:** Zbigniew Pozorski, Jolanta Pozorska, Ireneusz Kreja, Łukasz Smakosz

**Affiliations:** 1 Institute of Structural Engineering, Faculty of Civil and Transport Engineering, Poznan University of Technology, ul. Piotrowo 5, 60-965 Poznań, Poland; 2 Department of Mathematics, Faculty of Mechanical Engineering and Computer Science, Czestochowa University of Technology, Armii Krajowej 21, 42-201 Częstochowa, Poland; jolanta.pozorska@pcz.pl; 3 Department of Structural Mechanics, Faculty of Civil and Environmental Engineering, Gdańsk University of Technology, ul. Gabriela Narutowicza 11/12, 80-233 Gdańsk, Poland; ikreja@pg.edu.pl (I.K.); lukasz.smakosz@pg.edu.pl (Ł.S.)

**Keywords:** sandwich panels, local instability, strain energy, wrinkling, orthotropic core

## Abstract

This paper deals with the local loss of stability (wrinkling) problem of a thin facing of a sandwich panel. Classical solutions to the problem of a facing instability resting on a homogeneous and isotropic substructure (a core) are compared. The relations between strain energy components associated with different forms of core deformations are discussed. Next, a new solution for the orthotropic core is presented in detail, which is consistent with the classic solution for the isotropic core. Selected numerical examples confirm the correctness of the analytical formulas. In the last part, parametric analyses are carried out to illustrate the sensitivity of wrinkling stress to a change in the material parameters of the core. These analyses illustrate the possibility of using the equations derived in the article for the variability of Poisson’s ratio from −1 to 1 and for material parameters strongly deviating from isotropy.

## 1. Introduction

In a typical sandwich element, the two facings are joined to each other by a relatively thick but deformable core. The deformations and stresses in the sandwich panel are caused by the acting loads (wind, snow, self-weight, live load), but they are also largely due to thermal loads. As a result of these interactions, the facing can be compressed, and because it is connected to a susceptible substructure (a core), it very often experiences local loss of stability (wrinkling).

Wrinkling is undoubtedly one of the most common damage mechanisms of a sandwich element. For this reason, the correct estimation of the stress leading to the loss of facing stability is a key issue that has been undertaken by many researchers using various approaches: analytical, numerical, experimental, or mixed (or some combination of these approaches). Numerical methods allow for solving many complex problems, and the performed experiments allow for the verification of the obtained results. Nevertheless, analytical solutions should also be treated as very valuable, even if they are obtained with significant simplifications. Simple formulas are easy for engineering application and allow for a very quick (and continuous) assessment of the sensitivity of the solution to a change in design parameters.

With full awareness of the new challenges related to the sandwich structures (anisotropy [1], influence of extreme excitations [2], new production technologies [3], and many others), this work is an attempt to take a deeper look at the known classical solutions to the local instability problem [4,5,6]. The presented solution for an orthotropic core is based on the work of [7], in which sandwich columns under compression were considered, and the solution was presented in the form of hyperbolic functions. It also clearly refers to the classic solution for an isotropic core [6], where a facing and a core were assumed as infinite and the differential equation written for the facing was used.

The above-mentioned classic approaches to the problem of facing instability are constantly being used and extended to more and more complex issues. The analytical model that leads to wrinkling of the orthotropic face layer supported by a transversely isotropic core was presented in [8]. Wrinkling of a composite-facing sandwich panel under biaxial loading was discussed in [9]. Article [10] presents the solution to the symmetrical face sheet wrinkling problem using the energy method. The approach focused on a 3D case of wrinkling of orthotropic face sheets was presented in [11]. The analytical approach to the problem of anisotropic facing instability was presented in the works [12,13]. Wrinkling in sandwich structures with a functionally graded core was discussed in [14]. The papers [15,16,17] are examples of work on wrinkling, in which the core was modeled using higher-order theories, which allowed, among others, to take into account the influence of the core transverse compressibility.

This paper is divided into three parts. In the first one, we present some relations between the classical solutions to the analyzed problem of wrinkling. We believe that they will shed a slightly different light on known solutions. This applies to the conditions of reaching the critical stress, the influence of the Poisson ratio on the wrinkling stress, and the relationship between strain energy components. In the second part, the solution for the orthotropic core is derived and discussed, and we focus on the interpretation of the solution and the question of the conditions for obtaining it. In the third part, a parametric analysis of the solution for the orthotropic core is presented, illustrating the sensitivity of the solution (especially the wrinkling stress) to a change in some material parameters. In our opinion, this is essential for the optimal design of layered structures. By assuming certain constraints on material parameters, we can specify a solution with the maximum value or the minimum sensitivity.

## 2. Formulation of the Problem

We are considering a sandwich panel consisting of two thin facings and a thick but deformable core. Due to the bending of the composite panel, considerable compressive stresses may be generated in its facing, resulting in a local loss of stability. The instability has the form of wrinkling. In general, due to the variety of support and load conditions, the problem can be very complex; however, in practical civil engineering problems, a facing is usually compressed unidirectionally [18].

**Figure 1:**
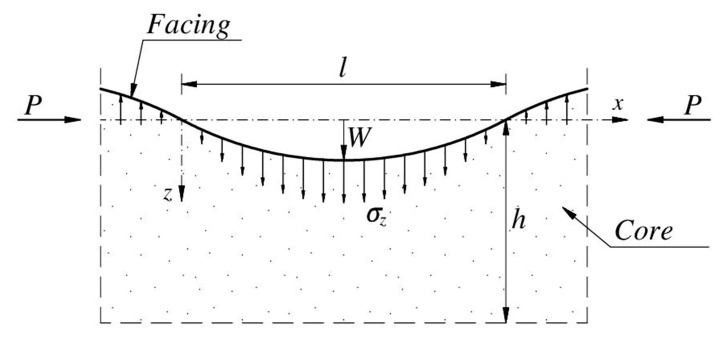
Assumed shape of a wrinkling.

The wrinkling phenomenon may be considered as a compression effect of a thin facing (treated as a beam or plate) supported by a continuous elastic core (Figure 1). The facing in tension is ignored because the deformation of the core quickly disappears as the distance from the compressed facing increases. It is convenient to assume that the compressed facing is infinitely long and the core extends to infinity on one side of the facing. The wrinkling is associated with short waves of buckling of the facing. Figure 1 shows a fragment of the deformed facing supported by the core.

It is assumed that the face layer is in a uniform stress and strain state. The deformations of the facing, which are infinite and periodic, induce strain and stress in the core. Core deformations quickly decay as the variable *z* increases, and the rate of this decay depends on the assumed displacement field.

The core and facing materials are homogeneous. Suppose the core is isotropic or orthotropic with one of the orthotropic axes coinciding with the direction of the compression. The facing material could be orthotropic if its axes were aligned with the material axes of the core. These are quite strong assumptions, but they give analytical results that are relatively easy to interpret.

## 3. Classical Solutions of the Wrinkling Problem

### 3.1. Energy Method—Linear Decay Function

Following the proposition of Hoff and Mautner [4], the core is affected only in a small zone with depth *h* (smaller than the thickness of the core). The shape of the face deformation is assumed in the sinusoidal form (Figure 1), and the core deformation field vanishes linearly with coordinate *z*:

(1) $$w_{C}=w_{F}\frac{\left(h-z\right)}{h}=W\frac{\left(h-z\right)}{h}\sin\frac{\pi x}{l},$$

where *w_C_*, *w_F_*, and *W* denote the vertical displacement of the core, face, and the displacement amplitude, respectively. The term *l* is a half wavelength of the wrinkles. Comparing the sum of strain energy of the core and the facing (per half wavelength) with the external work done by an applied work, the expression on the compressive stress in the facing is obtained:

(2) $$\sigma_{x}=\frac{E_{C}l^{2}}{\pi^{2}t_{F}h}+\frac{hG_{C}}{3t_{F}}+\frac{\pi^{2}E_{F}}{12}{\left(\frac{t_{F}}{l}\right)}^{2}.$$

Symbols *E_C_* and *G_C_* denote the modulus of elasticity and shear modulus of the isotropic core material, respectively. The thickness of the facing is *t_F_*, whereas the modulus of elasticity of the isotropic facing material is *E_F_*.

The minimum value of the compressive stress (2) corresponds to the critical (wrinkling) stress, and it can be found by using derivatives of *σ_x_* with respect to *h* and *l*:

(3) $$\sigma_{w}=\sqrt[{3}]{\frac{3}{4}}\cdot \sqrt[{3}]{E_{C}G_{C}E_{F}}\;≅\;0.909\cdot \sqrt[{3}]{E_{C}G_{C}E_{F}}.$$

It is worth noting that reaching the wrinkling stress corresponds to a situation in which each term on the right-hand side of Equation (2) is equal to each other.

### 3.2. Energy Method—Exponential Decay Function

Plantema [5] assumed the displacement field of the exponential form

(4) $$w_{C}=w_{F}e^{-kz}=We^{-kz}\sin\frac{\pi x}{l},$$

where *k* ≥ 0 is an auxiliary constant (with the unit inverse to the unit of variable *z*). The use of the strain energy of the core makes it possible to represent the compressive stress of the facing as

(5) $$\sigma_{x}=\frac{E_{C}kl^{2}}{2\pi^{2}t_{F}}+\frac{G_{C}}{2kt_{F}}+\frac{\pi^{2}E_{F}}{12}{\left(\frac{t_{F}}{l}\right)}^{2}.\;$$

The wrinkling stress is obtained from the conditions of zeroing the derivatives of *σ_x_* with respect to *k* and *l*. As we can see, a slightly different assumption of the displacement field leads to a different result. First of all, the condition for reaching the extreme (minimum) stress *σ_x_* is different. Again, when the critical stress is reached, each term of Equation (5) has the same value. The wrinkling stresses (3) and (6) are independent of the Poisson ratio of the core material.

(6) $$\sigma_{w}=\frac{3}{2\cdot \sqrt[{3}]{6}}\cdot \sqrt[{3}]{E_{C}G_{C}E_{F}}≅0.825\cdot \sqrt[{3}]{E_{C}G_{C}E_{F}}$$

### 3.3. Differential Equation Method

The solution based on the differential equation method was presented by Allen [6]. Stresses in the elastic isotropic medium can be defined using the Airy stress function *F* (*x*,*z*). The strain compatibility in the *x*–*z* plane leads to the bi-harmonic differential equation.

(7) $$\frac{\partial^{4}F}{\partial z^{4}}+2\frac{\partial^{4}F}{\partial x^{2}\partial z^{2}}+\frac{\partial^{4}F}{\partial x^{4}}=0.$$

Equation (7) is satisfied by the function

(8) $$F\left(x,z\right)=A\sin\frac{\pi x}{l}\left(1-Bz\right)e^{-\frac{\pi z}{l}},$$

where *A* and *B* are constants. Constant *B* can be found by using the condition that the *x*-displacements and strains at the surface of the core (*z* = 0) are equal to zero. Constant *A* can be expressed by the amplitude *W* of the *z*-displacement at *z* = 0. By using Allen’s method, nearly the entire mechanical field is obtained, which depends on *x* and *z* variables. If the state of plane stress is assumed, then displacements *u*, *v*, and *w*, strains *ε_x_*, *ε_y_*, *ε_z_*, and *γ_xz_*, and stresses *σ_x_*, *σ_y_*, and *τ_xz_* are non-zero.

The equilibrium differential equation for the facing has the form

(9) $$B_{F}\frac{d^{4}w}{dx^{4}}+P\frac{d^{2}w}{dx^{2}}=\sigma_{z},$$

where the stress *σ_z_* is the effect of the interaction between the facing and the core (see Figure 1). The symbol *B_F_* denotes the face bending stiffness per unit width. For the beam theory (as used here), $B_{F}=E_{F}t_{F}^{3}/12$; in the case of the plate theory, $B_{F}=E_{F}t_{F}^{3}/12\left(1-\nu_{F}^{2}\right)$. Using the function of the facing displacement

(10) $$w=W\sin\frac{\pi x}{l},$$

and the parameter *m* = *l*/*t_F_*, the compressive stress in the facing can be expressed as

(11) $$\sigma_{x}=\frac{\pi^{2}E_{F}}{12m^{2}}+\frac{a}{\pi }m=\sigma_{1}+\sigma_{2},$$

where

(12) $$a=\frac{2E_{C}}{\left(1+\nu_{C}\right)\cdot \left(3-\nu_{C}\right)}$$

is the material constant. The two terms of the solution for (11) are denoted as *σ*_1_ and *σ*_2_, respectively.

From the condition for the extreme, d*σ_x_*/d*m* = 0, we can find $m=\pi \cdot \sqrt[{3}]{E_{F}/6a}$ and the minimum critical (wrinkling) stress:

(13) $$\sigma_{w}=\sqrt[{3}]{\frac{9}{2\left(1+\nu_{C}\right)\cdot {\left(3-\nu_{C}\right)}^{2}}}\cdot \sqrt[{3}]{E_{C}G_{C}E_{F}}=r\cdot \sqrt[{3}]{E_{C}G_{C}E_{F}}.$$

If we assume the facing stiffness as for the plate, $B_{F}=E_{F}t_{F}^{3}/12\left(1-\nu_{F}^{2}\right)$, the modulus *E_F_* should be replaced by $E_{F}/\left(1-\nu_{F}^{2}\right)$.

It is interesting that for the minimum value of *σ_x_* (11), the second expression (*σ*_2_) is exactly two times higher than the first (*σ*_1_) [19]. From some literature sources, e.g., [6] p. 159, Figure 8.3, it can be drawn incorrectly that both of these values are equal. The value of the first root (*r*) depends only on the Poisson ratio of the core material *ν_C_*, but for the typical range of this parameter, the root *r* reaches the value from 0.780 to 0.794. It is also worth noting that as the Poisson ratio tends to −1, the critical stress would increase to infinity, although this is a rather theoretical situation.

### 3.4. Comparison of Classical Solutions

#### 3.4.1. Influence of the Poisson Ratio

Let us return first to Allen’s solution. The result (13) was obtained for a plane stress state. Assuming a plane strain state, the procedure is analogous; however, the functions of stresses, strains, and displacements are different. Equation (11) is valid, but:

(14) $$a=\frac{2E_{C}\left(1-\nu_{C}\right)}{\left(1+\nu_{C}\right)\cdot \left(3-4\nu_{C}\right)},$$

(15) $$\sigma_{w}=\sqrt[{3}]{\frac{9{\left(1-\nu_{C}\right)}^{2}}{2\left(1+\nu_{C}\right)\cdot {\left(3-4\nu_{C}\right)}^{2}}}\cdot \sqrt[{3}]{E_{C}G_{C}E_{F\;}}=s\cdot \sqrt[{3}]{E_{C}G_{C}E_{F}}.$$

**Figure 2:**
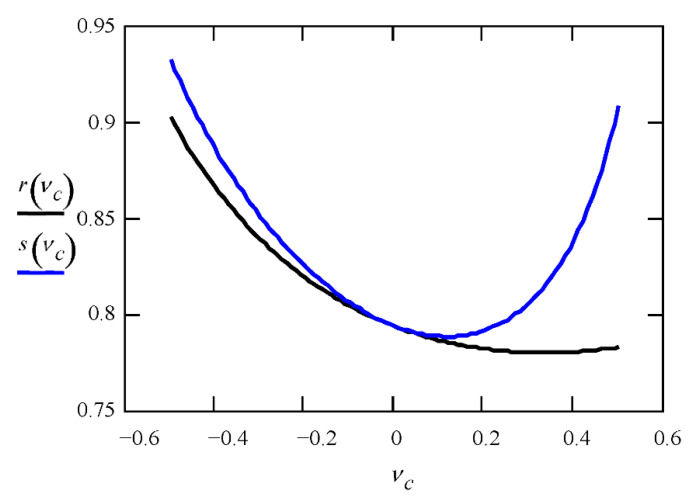
Comparison of Allen’s solution in the case of the plane stress (*r*) and plane strain (*s*) states.

Of course, the value of *s* in (15) is different than *r* in (13). To compare Allen’s solutions in the case of the plane stress and plane strain states, see Figure 2.

It should come as no surprise that for *v_C_* = 0, the coefficients *r* and *s* are identical and equal to 0.794. For negative values of *v_C_*, the coefficients *r* and *s* take similar values that are much higher than 0.794. For *v_C_* tending to −1, the values of *r* and *s*, and hence the critical stress values, tend to infinity. In the range of *v_C_* (−1; +0.5), the critical stresses in the plane strain state are higher than in the plane stress state, but the greatest differences between *r* and *s* appear for *v_C_* close to 0.5. This is obvious because in a plane state of stress, the material has the potential to deform in the *y*-direction (perpendicular to the plane), which facilitates the deformation of the facing. The plane strain condition limits the deformation (in the *y*-direction) and makes it difficult to buckle the facing. The greater the Poisson ratio, the greater the significance of this effect. For some order, let us remind you that the Poisson ratio of the core material does not affect the critical stresses in the case of the solutions given by Hoff and Mautner (3) and Plantema (6).

#### 3.4.2. Assumptions and Strain Energy Considerations

The Hoff–Mautner and Plantema solutions are based on an energy approach. The assumption of a specific displacement field turns out to be very effective and quickly leads to a solution. However, it is worth noting that in contrast to Allen’s solution, the assumed displacement fields ((1) or (4)) result in non-fulfillment of most of the differential equilibrium equations of a solid (mass forces were omitted in Equation (16)):

(16) $$\sigma_{ji,j}=0.$$

Let us return to the solution presented by Allen [6]. In the case of a plane stress state, constant *B* is

(17) $$B=-\frac{\pi }{2l}\left(1+\nu_{C}\right),$$

and the stresses in the core are expressed as the corresponding derivatives of the function *F* (*x*,*z*). By using commonly known physical and geometric relationships, we determine the fields of strain and displacement. Therefore, we can calculate the appropriate components of the strain energy of the core, obtaining, respectively:

(18) $$\frac{1}{2}\int_{0}^{\infty }\int_{0}^{l}\sigma_{x}\varepsilon_{x}dxdz=\frac{1}{16}\frac{A^{2}}{E_{C}}\frac{\pi^{3}}{l^{2}}\left(1+\nu_{C}\right)\left(1-\nu_{C}^{2}\right),$$

(19) $$\frac{1}{2}\int_{0}^{\infty }\int_{0}^{l}\sigma_{z}\varepsilon_{z}dxdz=\frac{1}{16}\frac{A^{2}}{E_{C}}\frac{\pi^{3}}{l^{2}}\left(1+\nu_{C}\right)\left(13-4\nu_{C}-\nu_{C}^{2}\right),$$

(20) $$\frac{1}{2}\int_{0}^{\infty }\int_{0}^{l}\tau_{xz}\gamma_{xz}dxdz=\frac{1}{16}\frac{A^{2}}{E_{C}}\frac{\pi^{3}}{l^{2}}\left(1+\nu_{C}\right)\left(10-4\nu_{C}+2\nu_{C}^{2}\right).$$

For *v_C_* = 0, the ratio of energies expressed in (18)–(20) is 1:13:10. Let us recall that under the condition of loss of stability, the elastic energy in the facing is half of the elastic energy in the core. Commenting on the relations between the energies in the core, we can say that the share of energy (18) resulting from the deformation of the core along the *x*-direction (*ε_x_*) is small, which can justify the omission of this term in classical energy methods. For the sake of order, we note that the fulfillment of the condition of loss of local stability for each of the previously discussed classical energy methods means that the energy components on the left side of Equations (19) and (20) are equal to each other, and the integral (18) is equal to zero.

An additional point requires clarification. Each of the presented classical methods differs in the final result, but only because of different assumptions and not because of the method itself. For example, this is easily demonstrated by using Allen’s mechanical fields for the energy approach. Then, it turns out that the obtained expression for the critical stress is identical to (13).

**Figure 3:**
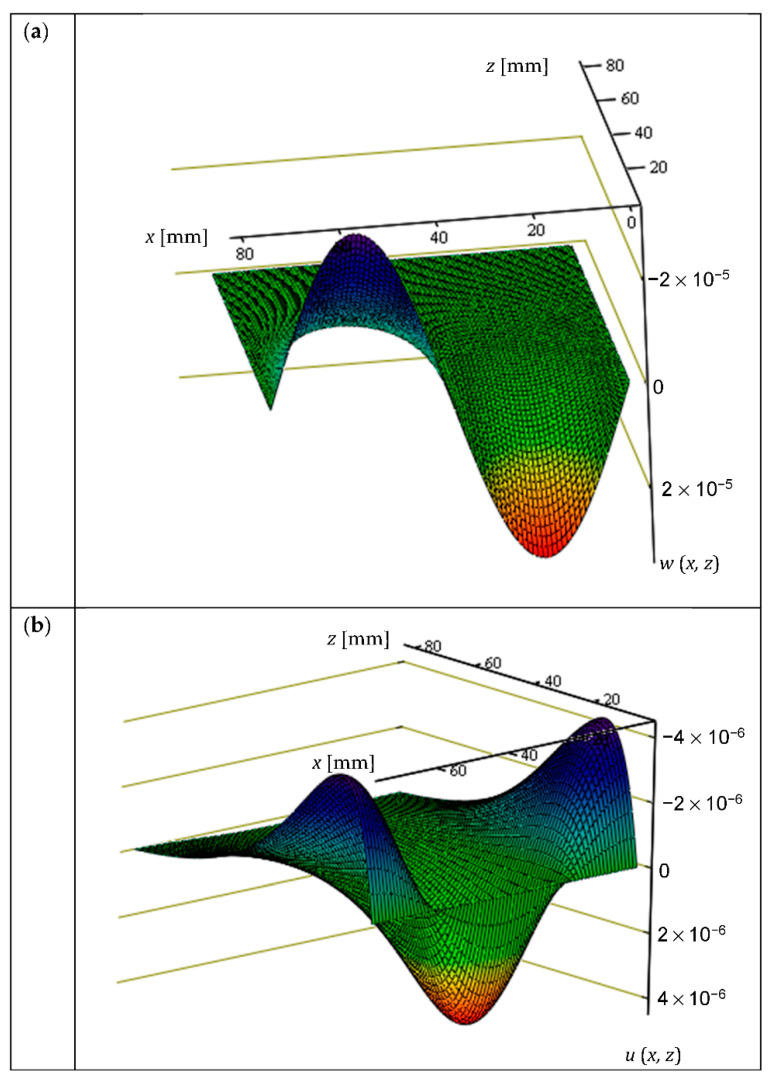
Solution obtained by using the differential equation method: (**a**) vertical displacement *w*(*x*, *z*), (**b**) horizontal displacement *u*(*x*, *z*).

For an illustration of the assumptions made in Allen’s solution, Figure 3 presents the displacement fields *w*(*x*, *z*) (perpendicular to the facing i.e., along the *z*-axis) and *u*(*x*, *z*) (along the *x*-axis). The values are given assuming the constant *A* = 1, see (8). The isotropic material of the core *E_C_* = 4 MPa, *v_C_* = 0.05 and a facing with a thickness of *t_F_* = 0.5 mm made of an isotropic material *E_F_* = 210 GPa were assumed. The range of the *x*-axis corresponds to 2*l* = 74 mm, while the *z*-coordinates are given in millimeters. In Figure 3a, for *z* = 0, we can see a full sinusoid with an extreme equal to 3.248 × 10^−5^, which disappears with increasing *z*. The amplitude of the sinusoid decreases 10 times for *z* = 36 mm. In Figure 3b, according to the assumption, for *z* = 0, the horizontal displacements are equal to zero. The variability of the function *u*(*x*, *z*) in the *x*-direction is described by the cosine function, the extreme value of 4.253 × 10^−6^ is reached for *z* = 12 mm; at a distance of *z* = 60 mm, the function value is 10 times smaller than the extreme. The rapid disappearance of displacements with the increment of the *z*-coordinate, and the values of *w*(*x*, *z*) being one order greater than *u*(*x*, *z*), are both noteworthy, as they, among other things, justify the omission of longitudinal deformations in classical energy methods.

## 4. Solution for the Orthotropic Core

### 4.1. Differential Equation

A certain solution to the problem of facing wrinkling resting on an orthotropic elastic substructure and loaded on the edge (in the facing plane) was presented in [7]. A similar approach was used in [8]. The following is a detailed solution, which is an extension of [6], formally based on [7,8], but it differs in some nuances. Efforts were made to present the solution precisely in order to also discuss the conditions for obtaining this solution.

Suppose we have an orthotropic core, in which orthotropic axes coincide with the axes of the element. The facing is compressed uniaxially, and the load direction is according to the material axes of the core. Such a situation is very common in practice [20]. The constitutive relation for the orthotropic core material is:

(21) $$\left\{\begin{array}{c} \begin{array}{c} \varepsilon_{x}\\ \varepsilon_{y}\\ \varepsilon_{z} \end{array}\\ \varepsilon_{xy}\\ \begin{array}{c} \varepsilon_{xz}\\ \varepsilon_{yz} \end{array} \end{array}\right\}=\left[\begin{array}{cccccc} 1/E_{x} & -\nu_{yx}/E_{y} & -\nu_{zx}/E_{z} & 0 & 0 & 0\\ -\nu_{xy}/E_{x} & 1/E_{y} & -\nu_{zy}/E_{z} & 0 & 0 & 0\\ -\nu_{xz}/E_{x} & -\nu_{yz}/E_{y} & 1/E_{z} & 0 & 0 & 0\\ 0 & 0 & 0 & 1/2G_{xy} & 0 & 0\\ 0 & 0 & 0 & 0 & 1/2G_{xz} & 0\\ 0 & 0 & 0 & 0 & 0 & 1/2G_{yz} \end{array}\right]\left\{\begin{array}{c} \begin{array}{c} \sigma_{x}\\ \sigma_{y}\\ \sigma_{z} \end{array}\\ \begin{array}{c} \tau_{xy}\\ \tau_{xz}\\ \tau_{yz} \end{array} \end{array}\right\}.$$

In the case of a 2D problem, relation (21) can be simplified to:

(22) $$\begin{array}{c} \varepsilon_{x}=a_{xx}\sigma_{x}-a_{xz}\sigma_{z}\;\\ \varepsilon_{z}=-a_{xz}\sigma_{x}+a_{zz}\sigma_{z}\\ \varepsilon_{xz}=\left(1/2G_{xz}\right)\tau_{xz} \end{array}\}.$$

In the case of plane stress state, material constants *a_xx_*, *a_zz_*, and *a_xz_* are:

(23) $$\begin{array}{c} a_{xx}=1/E_{x}\;\\ a_{zz}=1/E_{z}\\ a_{xz}=\nu_{xz}/E_{x} \end{array}\},$$

whereas for the plane strain state, we have:

(24) $$\begin{array}{c} a_{xx}=\frac{1-\nu_{xy}\nu_{yx}}{E_{x}\;}\;\\ a_{zz}=\frac{1-\nu_{yz}\nu_{zy}}{E_{z}\;}\\ a_{xz}=\frac{\nu_{xz}+\nu_{xy}\nu_{yz}}{E_{x}\;} \end{array}\}.$$

The compatibility of strains in the *x–z* plane requires:

(25) $$\frac{\partial^{2}\varepsilon_{x}}{\partial z^{2}}+\frac{\partial^{2}\varepsilon_{z}}{\partial x^{2}}\;–\;2\frac{\partial^{2}\varepsilon_{xz}}{\partial x\partial z}=0.$$

After introducing the Airy stress function *F* (*x*, *y*) such that

(26) $$\sigma_{x}=\frac{\partial^{2}F}{\partial z^{2}},\;\sigma_{z}=\frac{\partial^{2}F}{\partial x^{2}},\;\tau_{xz}=-\frac{\partial^{2}F}{\partial x\partial z},$$

condition (25) takes the following form:

(27) $$a_{zz}\frac{\partial^{4}F}{\partial x^{4}}+2\left(\frac{1}{2G_{xz}}-a_{xz}\right)\frac{\partial^{4}F}{\partial x^{2}\partial z^{2}}+a_{xx}\frac{\partial^{4}F}{\partial z^{4}}=0.$$

By using substitution

(28) $$\eta =\epsilon z=\left(\sqrt[{4}]{a_{zz}/a_{xx}}\right)z,$$

we obtain

(29) $$\frac{\partial^{4}F\left(x,\eta \right)}{\partial x^{4}}+2\kappa \frac{\partial^{4}F\left(x,\eta \right)}{\partial x^{2}\partial \eta^{2}}+\frac{\partial^{4}F\left(x,\eta \right)}{\partial \eta^{4}}=0,$$

where *κ* is a dimensionless quantity and depends only on the material parameters of the core,

(30) $$\kappa =\frac{1}{\sqrt{a_{xx}a_{zz}}}\left(\frac{1}{2G_{xz}}\;–\;a_{xz}\right).$$

For an isotropic material, *κ* = 1.

### 4.2. Solution of the Differential Equation

To find a solution of (29), we separate variables:

(31) F(x, η)=G(x)H(η)

and assume the sinusoidal form of function *G* (*A*_1_ is a constant)

(32) $$G\left(x\right)=A_{1}\sin\frac{\pi x}{l}$$

which leads to

(33) $$\frac{d^{4}H}{dx^{4}}-2\kappa {\left(\frac{\pi }{l}\right)}^{2}\frac{d^{2}H}{d\eta^{2}}+{\left(\frac{\pi }{l}\right)}^{4}H=0.$$

By assuming that the function *H*(*η*) = *e^λη^* is a general solution of Equation (33), we obtain a solution in the form of a linear combination of this function:

(34) $$H\left(\eta \right)=C_{1}e^{\lambda_{1}\eta }+C_{2}e^{\lambda_{2}\eta }+C_{3}e^{\lambda_{3}\eta }+C_{4}e^{\lambda_{4}\eta },$$

where

(35) $$\begin{array}{cc} \lambda_{1}=+\frac{\pi }{l}\sqrt{\kappa -\sqrt{\kappa^{2}-1}}\;, & \lambda_{2}=-\frac{\pi }{l}\sqrt{\kappa -\sqrt{\kappa^{2}-1}}\\ \lambda_{3}=+\frac{\pi }{l}\sqrt{\kappa +\sqrt{\kappa^{2}-1}}\;, & \lambda_{4}=-\frac{\pi }{l}\sqrt{\kappa +\sqrt{\kappa^{2}-1}} \end{array}\}.$$

The positive solutions for *λ* have to disappear to allow an exponential decrease in the stresses in the thickness direction *z*. Therefore, *C*_1_ = 0, *C*_3_ = 0, and

(36) $$F\left(x,\;y\right)=\left[C_{2}e^{-\frac{\pi }{l}\sqrt{\kappa -\sqrt{\kappa^{2}-1}}\epsilon z}+C_{4}e^{-\frac{\pi }{l}\sqrt{\kappa +\sqrt{\kappa^{2}-1}}\epsilon z}\right]A_{1}\sin\frac{\pi x}{l}=\;\;\;\;\;\;\;\left[B_{1}e^{-\frac{\pi }{l}\sqrt{\kappa -\sqrt{\kappa^{2}-1}}\epsilon z}+B_{2}e^{-\frac{\pi }{l}\sqrt{\kappa +\sqrt{\kappa^{2}-1}}\epsilon z}\right]\sin\frac{\pi x}{l},$$

where *B*_1_ = *C*_2_ *A*_1_ and *B*_2_ = *C*_4_ *A*_1_ are constants. These constants can be calculated with the following boundary conditions:

(37) $$\varepsilon_{x}\left(z=0\right)=0,$$

which reflects the observation that the face material is typically much stiffer than the core material, and

(38) $$\sigma_{z}\left(z=0\right)=A\sin\frac{\pi x}{l},$$

because the stress at the interface in the *z*-direction is distributed as a sine wave with a certain amplitude *A* corresponding to the assumed wave deformation. From the assumed boundary conditions, we obtain:

(39) $$\{\begin{array}{c} B_{1}=-A{\left(\frac{l}{\pi }\right)}^{2}\frac{a_{xz}+a_{xx}\epsilon^{2}\left(\kappa +\sqrt{\kappa^{2}-1}\right)}{2a_{xx}\epsilon^{2}\sqrt{\kappa^{2}-1}}\\ B_{2}=A{\left(\frac{l}{\pi }\right)}^{2}\frac{a_{xz}+a_{xx}\epsilon^{2}\left(\kappa -\sqrt{\kappa^{2}-1}\right)}{2a_{xx}\epsilon^{2}\sqrt{\kappa^{2}-1}} \end{array}.$$

It is easy to note that $B_{1}+B_{2}=-A{\left(\frac{l}{\pi }\right)}^{2}$.

The equilibrium differential equation for the facing has the same form as (9). By integrating *ε_z_* (22), we can find the following expression for the facing displacement *w_F_* = *w*(*z* = 0)

(40) $$w\left(z=0\right)=\frac{\pi }{l}\sin\frac{\pi x}{l}\left[a_{xz}B_{1}\epsilon \sqrt{\kappa -\sqrt{\kappa^{2}-1}}+a_{xz}B_{2}\epsilon \sqrt{\kappa +\sqrt{\kappa^{2}-1}}+\;\;\;\;\;\;\;\;\;\;\;\;\;\;\;\;\;\;\;a_{zz}B_{1}\frac{1}{\epsilon \sqrt{\kappa -\sqrt{\kappa^{2}-1}}}+a_{zz}B_{2}\frac{1}{\epsilon \sqrt{\kappa +\sqrt{\kappa^{2}-1}}}\right].$$

By performing some additional algebraic transformations as suggested by Allen [6], one can arrive at the analogy of (11) with

(41) $$a=\frac{\sqrt{\kappa^{2}-1}}{\sqrt{\kappa +\sqrt{\kappa^{2}-1}}-\sqrt{\kappa -\sqrt{\kappa^{2}-1}}}\frac{2a_{xx}\epsilon }{2a_{xx}a_{xz}\epsilon^{2}-a_{xz}^{2}+a_{xx}a_{zz}\left(2\kappa +1\right)}.$$

From the condition for the extreme d*σ_x_*/d*m* = 0, we have as before

(42) $$m=\pi \cdot \sqrt[{3}]{\frac{E_{F}}{6a}}$$

and the minimum critical (wrinkling) stress is obtained (the solution is consistent with (13)):

(43) $$\sigma_{w}=\frac{3}{2\sqrt[{3}]{6}}\cdot \sqrt[{3}]{a^{2}E_{F}}.$$

**Figure 4:**
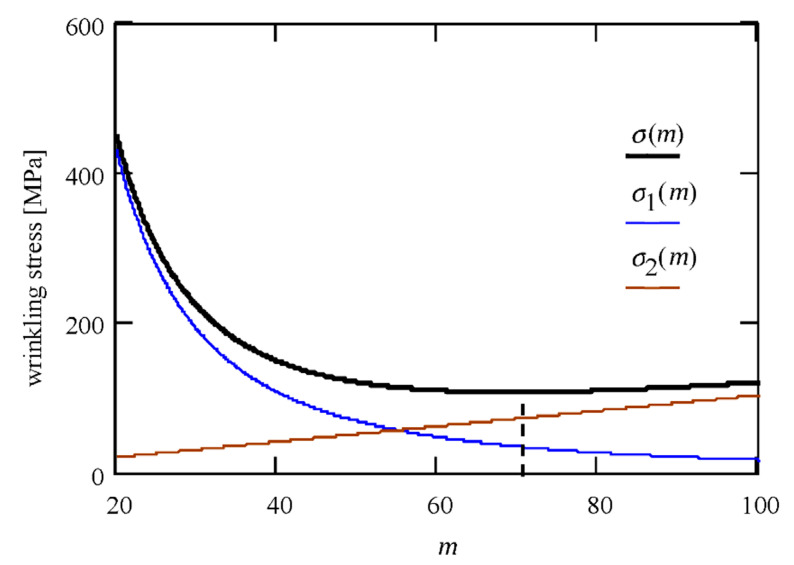
Wrinkling stress as a function of *m* parameter.

It is easy to prove that again, *σ*_2_ = 2*σ*_1_ (see also Figure 4).

Based on the quick analysis of Equation (41), it can be concluded that for *a*, which has the nature (and the measurement unit) of the stiffness modulus (of the core), the condition *κ* > 1 must be satisfied to reach real values. With smaller values of *κ*, the roots in Equation (41) are complex numbers. However, it is somewhat surprising that despite the complex roots in (41), the value of *a*,

(44) $$a=\sqrt{\frac{\kappa +1}{2}}\frac{2a_{xx}\epsilon }{2a_{xx}a_{xz}\epsilon^{2}-a_{xz}^{2}+a_{xx}a_{zz}\left(2\kappa +1\right)},$$

is real if the condition *κ* > −1 is satisfied. In order for parameter *m* to be positive, the denominator in expression (41) must be positive (the nominator is positive). When the denominator in (41) approaches 0^+^, *a* and consequently also *σ_w_* tend to infinity.

Let us take a moment to analyze the value of *κ* in a plane stress state. According to (30), we have

(45) $$\kappa =\sqrt{E_{x}E_{z}}\left(\frac{1}{2G_{xz}}-\frac{\nu_{xz}}{E_{x}}\right).$$

Modules *E_x_*, *E_z_*, and *G_xz_* must be positive. In this situation, if *ν_xz_* is negative, then *κ* will always be positive. If *ν_xz_* = 0.5, then *κ* is positive when *E_x_* > *G_xz_*; if *ν_xz_* = 1, then *κ* is positive when *E_x_* > 2*G_xz_*. Let us recall that in the case of orthotropic materials, the condition for the stability of the material behavior is not only the positive values of the *E_x_*, *E_z_*, and *G_xz_* modules, but also, among others [21],

(46) $$\left|\nu_{xz}\right|<\sqrt{E_{x}/E_{z}}.$$

## 5. Examples

### 5.1. Analytical Solutions

The first example concerns the facing with a thickness of *t_F_* = 0.5 mm made of an isotropic material (*E_F_* = 210 GPa) placed on an isotropic core (*E_C_* = 4 MPa, *ν_C_* = 0.05; therefore *G_C_* = *E_C_*/2(1 + *ν_C_* ) = 1.905 MPa). According to the approach of Hoff–Mautner (3), Plantema (6), and Allen (13), we will obtain the following wrinkling stresses, respectively: *σ^H-M^* = 106.32 MPa, *σ^P^* = 96.49 MPa, and *σ^A^* = 92.36 MPa.

Now let us consider the same facing (*t_F_* = 0.5 mm *E_F_* = 210 GPa) supported by an orthotropic substructure (*E_x_* = 10 MPa, *E_z_* = 4 MPa, *ν_xz_* = 0.05, *G_xz_* = 3 MPa).

We assume a plane state of stress in the *x–z* plane and look for the critical stress that will cause the wrinkling of the facing. According to (28), (30), and (41), we will get ϵ = 1.257, *κ* = 1.022, and *a* = 3.256 MPa. The wrinkling stress is achieved for *m* = 69.336 (see (42)) and according to (43), *σ_w_* = 107.8 MPa. Figure 4 shows the dependence of the critical stress (solid line) on the *m* parameter. The blue and brown lines show both stress components (11).

### 5.2. Numerical Solutions

Numerical analysis of the instability problems of all kinds of structures is an intriguing and fascinating task, but it is not easy. First of all, it should be realized that numerical models are often much more complex than analytical models. This is due to the fact that commercial software (using, for example, the finite element method) allows for a relatively quick creation of spatial models. However, the problem is that the appropriate model class requires boundary conditions corresponding to this model. Therefore, these conditions are usually different than in the analytical model, which makes it difficult to compare the solutions. This issue was pointed out by numerous researchers [22,23,24]. This problem also arises when it comes to determining the critical stresses in a thin facing resting on a susceptible substructure.

The numerical analysis of the discussed issue was prepared using ABAQUS, which is a software suite for finite element analysis and computer-aided engineering. The problem was solved using two different classes of numerical models: 2D and 3D. A detailed description of the 3D model is presented below. The results obtained for the 2D model are presented at the end of the subsection.

**Figure 5:**
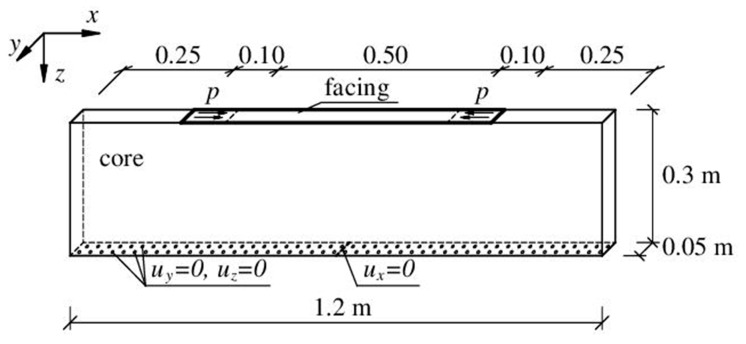
Numerical model of the problem of compression of a thin facing resting on a susceptible substructure.

The three-dimensional model was created in order to fully analyze the phenomenon of loss of stability in conditions close to the plane stress state. Of course, we also tried to make the numerical model as close as possible to the analytical model. The model space is not infinite, but the dimensions have been defined so that the displacements, strains, and stresses at the edge of the model are relatively small; the core body was 1.2 m long and 0.3 m high. The core thickness was 0.05 m, which should provide a freedom of deformation along the *y*-axis. A facing strip 0.5 mm thick and 0.7 m long rests on such a substructure. The geometry of the system is shown in Figure 5.

In order to compress the facing, an area of 0.10 m × 0.05 m was determined on its two ends, to which a uniform pressure *p* was applied in the *x*-direction (tangent to the facing, opposite at the two ends). The load application area is distant from the edge of the substructure (0.25 m). The decision was made to apply the load distributed over the surface because the attempt to load the system in the form of a linear load applied to the edge of the facing caused too much local disturbance. As the system had to be supported, after several attempts, it was decided to support the bottom surface of the substructure. The displacement conditions *u_y_* = 0, *u_z_* = 0 were assumed on the entire bottom surface, and additionally, *u_x_* = 0 was assumed in the middle of this surface, which is shown in Figure 5. This support had a small influence on the behavior of the system, while ensuring its necessary stabilization. When trying to limit the displacements on the sides of the substructure, it turned out that these limitations affect the behavior of the system and cause stress disturbances. The attempt to define the boundary conditions identical to those in the analytical model was unsuccessful. The assumption that the horizontal displacement of the facing equals zero made it practically impossible to induce the appropriate stress state in this facing. The description of the model shows that despite all efforts, the numerical model has some deviations from the theoretical model in which the core (substructure) is an infinite elastic half-space.

The material parameters of the 3D numerical model corresponded to the analytical model. The facing material was assumed to be isotropic elastic (*E_F_* = 210 GPa, *ν_F_* = 0.3). In the case of an isotropic core, it was assumed *E_C_* = 4 MPa, *ν_C_* = 0.05. When the case with the orthotropic core was analyzed, its parameters were defined as follows: *E_x_* = 10 MPa, *E_y_* = 10 MPa, *E_z_* = 4 MPa, *ν_xy_* = *ν_xz_* = *ν_yz_* = 0.05, *G_xy_* = *G_xz_* = *G_yz_* = 3 MPa. The 3D model uses C3D8 solid elements (core) and S4 shell elements (facing), in which there is no reduced integration. Interaction between the facing and the core was defined using a TIE connection, which causes the displacements of nodes of one surface to be identical to the displacements of nodes on the other surface. The size of the finite element mesh was constant and equal to 0.01 m. It is worth mentioning that the problem of facing wrinkling is mesh-dependent. The mesh should be dense enough to allow deformation of the core and facing. Thus, the mesh size is dependent on the finite element itself (a shape function) as well as the properties of the facing and core materials. In the case of S4 finite elements (doubly curved general purpose shell, finite membrane strains), it is sufficient if there are two finite elements per half-wavelength *l*. In our case, the half-wavelength was in the order of 0.035–0.038 m; therefore, the size of the finite elements turned out to be small enough (0.01 m).

Since the phenomenon of face wrinkling is associated with the local deformation of the compressed face, a geometrically nonlinear static analysis and the Riks method were used. Due to the symmetry of the problem, it turned out to be beneficial to introduce into the model’s initial imperfections as a linear combination of buckling modes of the structure. The buckling modes were solved independently. The size of the introduced imperfections was very small. The sum of four modes multiplied by 0.00001 was introduced, which meant that the positions of the model nodes were disturbed about 0.025 mm. This means that the amplitude of the imperfection was 5% of the facing thickness.

The load applied to the model could increase to the value of 2000 kPa, which corresponds to a compressive force of 10 kN and a compressive stress in the facing of 400 MPa. Obviously, such a load value was never realized because the facing had previously buckled. The applied load level was determined on the basis of the LPF (Load Proportionality Factor) value.

**Figure 6:**
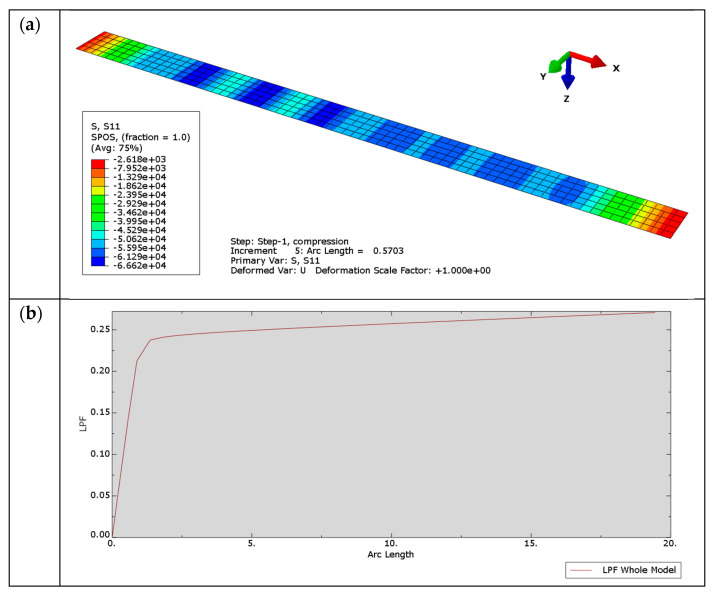
Numerical solution of the compression of the elastic facing resting on the susceptible core: (**a**) *σ_x_* stress at the top of the facing (fifth load increment) (**b**) LPF–arc length relation.

Another interesting challenge of numerical analysis is the question of recognizing when a structure loses stability and when it does not. Unfortunately, as in real conditions, and unlike in analytical solutions, in a numerical solution, there is usually no unambiguous parameter indicating the state of the system (stable–unstable). Wrinkles in the compressed facing appear very quickly, which is illustrated in Figure 6a (only the facing was presented). A certain determinant of instability may be the appearance of a nonlinear relationship between LPF and arc length factor (Figure 6b), indicating the nonlinear nature of the process [25]. One should also pay attention to the difference between the compressive stress in the facing in the *x*-direction calculated on the basis of the currently applied force divided by the facing cross-section area and the stress obtained in the FE model that takes into account nonlinear effects, i.e., local deformations. The comparison of these stresses for the first eight load increments is presented in Table 1. The stress values estimated at the FE nodes are much higher due to the effect of the load acting on the distance resulting from the deformation of the facing. Since the material was originally assumed to be perfectly elastic, the stresses in the model can be very high.

**Figure 7:**
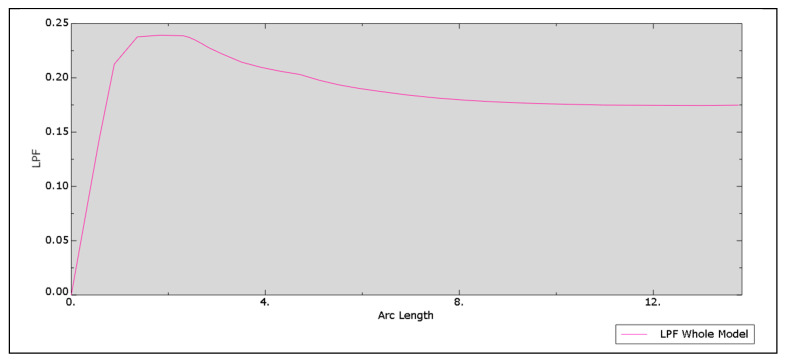
Numerical solution of the compression of the elastic–plastic facing resting on the susceptible core: LPF–arc length relation.

This situation, which is complex for evaluation, definitely changes after assuming that the facing material is perfectly elastic–plastic. Assuming the yield point *f_y_* = 270 MPa (the value is consistent with the characteristics of typical steel sheets used for the production of sandwich panels), in the ninth load increment, for LPF = 0.239, the LPF–arc length diagram breaks down (Figure 7), which corresponds to the theoretical facing compression stress 0.239 × 400 = 95.6 MPa.

A very similar relationship can be observed in the analysis of the problem with the orthotropic core. A breakdown of the LPF–arc length relationship occurred for LPF = 0.284, which corresponds to the theoretical stress 113.6 MPa. Of course, the stresses in the nodes of the model are different and reach the yield point of the material.

The obtained numerical results (95.6 MPa and 113.6 MPa) are close to the theoretical values (92.36 MPa and 107.8 MPa). Introduction of the yield stress for the facing material facilitates the interpretation of the numerical results. It is also worth paying attention to the fact that for the seventh or eighth load increment (Table 1), the stresses in the core reach the values close to the strength of typical core materials.

A number of numerical analyses were also carried out using the 2D model. The geometry and boundary conditions of this model corresponded to the geometry and boundary conditions of the 3D model. The main difference between the models was that they used plane (not spatial) finite elements: CPS4 for the core and B23 beam elements for the facing. It turned out that the 2D model behaves very similarly to the 3D model. Among other things, there are similar difficulties in interpreting the moment of loss of stability. This situation changes after assuming that the facing material is perfectly elastic–plastic. For the isotropic core, the LPF–arc length relationship is very similar to the relation presented in Figure 7, but the breakdown occurs for LPF = 0.227, which corresponds to the facing compression stress 0.227 × 400 = 90.8 MPa. For the orthotropic core, the extreme LPF value is 0.259, which corresponds to the stress of 103.6 MPa. These values are similar to the analytical results, although they are slightly lower than in the case of the 3D model.

## 6. Parametric Analysis

### Description of the Models

**Figure 8:**
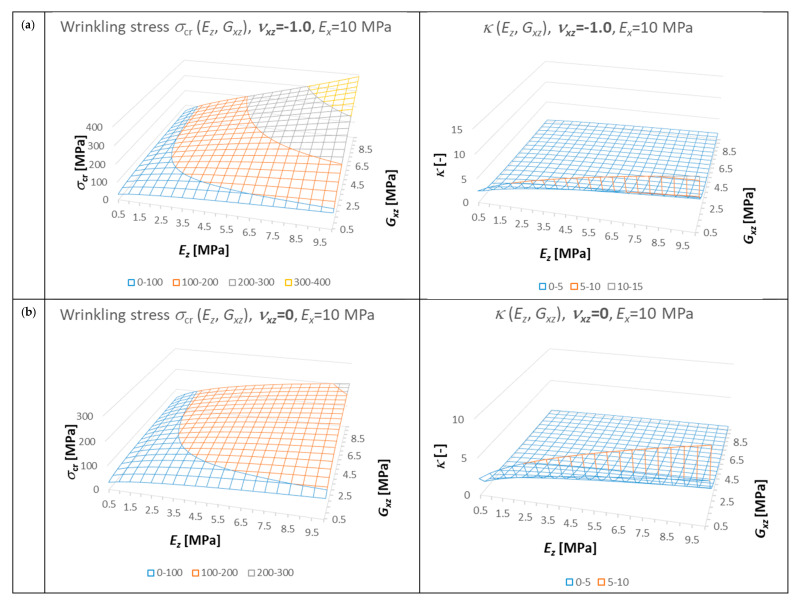

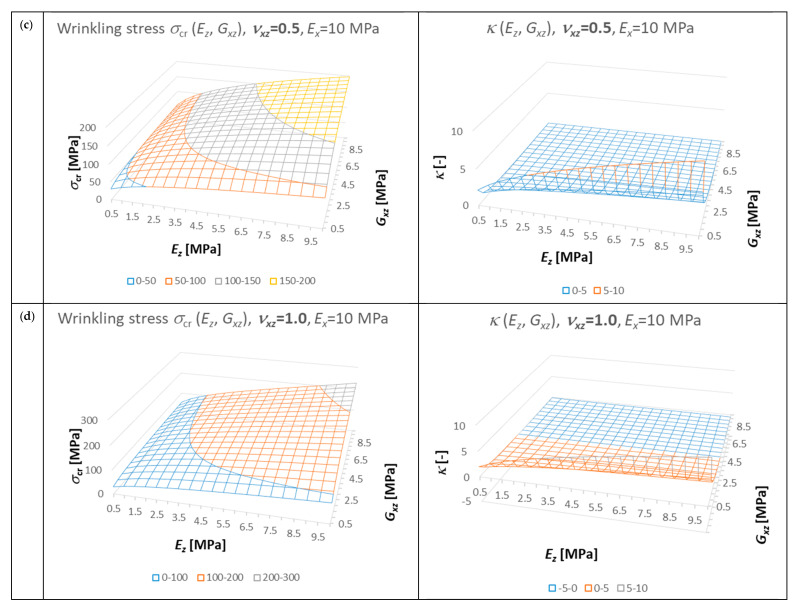
Influence of core material parameters on the value of wrinkling stress and the parameter *κ*: (**a**) *ν_xz_* = −1.0, (**b**) *ν_xz_* = 0, (**c**) *ν_xz_* = 0.5, (**d**) *ν_xz_* = 1.0.

Using the derived formulas, the influence of the material parameters of the orthotropic core on the value of the wrinkling stress was calculated and illustrated (Figure 8). Modulus *E_x_* = 10 MPa was assumed as constant. The modules *E_z_* and *G_xz_* are variable. Moreover, each of the graphs corresponds to a different value of the Poisson ratio *ν_xz_*, namely −1.0, 0.0, 0.5, and 1.0. For additional illustration of the problem, the graphs of the parameter *κ* are also presented in Figure 8.

The basic conclusions from the analysis of the graphs are quite obvious and consistent with the case of the isotropic core: the greater the stiffness of the core, the higher the wrinkling stress. It gets more interesting when *ν_xz_* = 1, because with large *E_z_* and *G_xz_* the parameter *κ* approaches −1 and the parameter *a* increases strongly. In the case when *ν_xz_* = −1, the parameter *κ* takes values in the typical range (positive values), but with large values of *E_x_* and *G_xz_*, the parameter *a* (44) reaches much higher values and grows faster than the parameter *m* drops (42). It should be emphasized that for the presented range of variability, *κ* > −1 and *a* is a positive value.

## 7. Conclusions

The first part of the article contained a short survey of the classical solutions to the problem of instability of a facing resting on a homogeneous and isotropic substructure (core). It was presented how the assumptions concerning the displacement field affect the solution of the problem. Next, the dependence of the solution [6] on the value of the Poisson ratio was presented, and strain energy analyses were carried out to investigate the relationships between the individual components of the deformation energy of the core. In the second part of the paper, the derivation of the formula for the critical stress in the case of uniaxial compression of the thin facing resting on the orthotropic core was presented. The conditions for the existence of the solution were discussed, which in principle are met for a wide range of variability of material parameters. The numerical example confirming the compliance of the selected analytical solution with the numerical one was also presented. The article discussed the applied models in detail and explained the difficulties associated with determining the load and support boundary conditions. The presented numerical model has not been experimentally verified, although a similar model was verified in [19] for a core with the Poisson ratio ranging from 0 to 0.3. The third part of the article presented the results of the parametric analysis, i.e., the effect of changing the material parameters of the orthotropic core on the wrinkling stress. This type of analysis can be of great importance in the optimal design of sandwich systems where local loss of stability plays a significant role. The developed solution can be easily introduced into the optimization procedure.

The presented work confirms that the further development of analytical methods in solving the discussed problem is advisable and important, both from a scientific and engineering point of view. Undoubted benefits also come from the possibility of numerical analysis of the issue under discussion. The applied FE models revealed that due to the local loss of stability, the stresses in the facing locally increase to the yield point, and the stresses in the core reach values similar to the strength of the core material. This means that if we want to accurately understand the stress state in the facing and the core, a relatively simple and attractive analytical approach should be supplemented with a numerical solution.

## Figures and Tables

**Table 1:** The comparison of compressive stress in the facing for the load increments of the numerical model.

Load Increment	The Percentage Completion of the Load Step (LPF)	Theoretical Compressive Stress *σ_x_* [MPa]	Extreme Compressive Stress Read in the Model Nodes *σ_x_* [MPa]
1	0.0156	6.24	6.72
2	0.0313	12.52	13.52
3	0.0547	21.88	23.88
4	0.0898	35.92	39.99
5	0.142	56.80	66.62
6	0.213	85.20	129.5
7	0.238	95.20	263.8
8	0.241	96.40	330.4

## Data Availability

The data presented in this study are available on request from the corresponding author.

## References

[1] Vescovini R., D’Ottavio M., Dozio L., Polit O. (2018). Buckling and wrinkling of anisotropic sandwich plates. Int. J. Eng. Sci..

[2] Studziński R., Gajewski T., Malendowski M., Sumelka W., Al-Rifaie H., Peksa P., Sielicki P.W. (2021). Blast test and failure mechanisms of soft-core sandwich panels for storage halls applications. Materials.

[3] Zaharia S.M., Enescu L.A., Pop M.A. (2020). Mechanical performances of lightweight sandwich structures produced by material extrusion-based additive manufacturing. Polymers.

[4] Hoff N.J., Mautner S.E. (1945). Buckling of Sandwich Type Panels. J. Aeronaut. Sci..

[5] Plantema F.J. (1966). Sandwich Construction; the Bending and Buckling of Sandwich Beams, Plates and Shells.

[6] Allen H.G. (1969). Analysis and Design of Structural Sandwich Panels.

[7] Norris C.B., Ericksen W.S., March H.W., Smith C.B., Boller K.H. (1961). Wrinkling of the Facing of Sandwich Construction Subjected to Edgewise Compression, Report No. 1810.

[8] Vonach W.K., Rammerstorfer F.G. (2000). Wrinkling of thick orthotropic sandwich plates under general loading conditions. Arch. Appl. Mech..

[9] Birman V., Bert C.W. (2004). Wrinkling of composite-facing sandwich panels under biaxial loading. J. Sandw. Struct. Mater..

[10] Lopatin A., Morozov E. (2008). Symmetrical facing wrinkling of composite sandwich panels. J. Sandw. Struct. Mater..

[11] Koissin V., Shipsha A., Skvortsov V. (2011). Wrinkling in sandwich panels—An analytical approach. J. Sandw. Struct. Mater..

[12] Fagerberg L. (2003). The effect of local bending stiffness on wrinkling of sandwich panels. J. Eng. Marit. Environ..

[13] Fagerberg L., Zenkert D. (2005). Effects of anisotropy and multiaxial loading on the wrinkling of sandwich panels. J. Sandw. Struct. Mater..

[14] Birman V., Vo N. (2017). Wrinkling in sandwich structures with a functionally graded core. J. Appl. Mech..

[15] Frostig Y. (2011). On wrinkling of a sandwich panel with a compliant core and self-equilibrated loads. J. Sandw. Struct..

[16] Hohe J., Librescu L. (2008). Recent results on the effect of the transverse core compressibility on the static and dynamic response of sandwich structures. Composites Part B Eng..

[17] Phan C.N., Bailey N.W., Kardomateas G.A., Battley M.A. (2012). Wrinkling of sandwich wide panels/beams based on the extended high-order sandwich panel theory: Formulation, comparison with elasticity and experiments. Arch. Appl. Mech..

[18] (2013). EN 14509:2013 Self-Supporting Double Skin Metal Faced Insulating Panels—Factory Made Products—Specifications.

[19] Pozorski Z. (2016). Sandwich Panels in Civil Engineerin—Theory, Testing and Design.

[20] Garbowski T., Gajewski T., Grabski J.K. (2020). Torsional and transversal stiffness of orthotropic sandwich panels. Materials.

[21] Lempriere B.M. (1968). Poisson’s Ratio in Orthotropic Materials. AIAA J..

[22] Steeves C.A., Fleck N.A. (2004). Collapse mechanisms of sandwich beams with composite faces and a foam core, loaded in three-point bending. Part II: Experimental investigation and numerical modelling. Int. J. Mech. Sci..

[23] Smakosz Ł., Kreja I., Pozorski Z. (2020). Flexural behavior of composite structural insulated panels with magnesium oxide board facings. Arch. Civ. Mech. Eng..

[24] Pozorski Z., Wojciechowski S. (2020). The influence of symmetrical boundary conditions on the structural behaviour of sandwich panels subjected to torsion. Symmetry.

[25] Riks E. (1979). An incremental approach to the solution of snapping and buckling problems. Int. J. Solids Struct..

